# Variation on gut microbiota diversity of endangered red pandas (*Ailurus fulgens*) living in captivity acrosss geographical latitudes

**DOI:** 10.3389/fmicb.2024.1420305

**Published:** 2024-08-06

**Authors:** Wenqi Chen, Xiaobing Chen, Yushuo Zhang, Hong Wu, Dapeng Zhao

**Affiliations:** Tianjin Key Laboratory of Conservation and Utilization of Animal Diversity, College of Life Sciences, Tianjin Normal University, Tianjin, China

**Keywords:** endangered species, health care, *ex situ* conservation, environmental influence, intestinal flora

## Abstract

The gut microbiome plays important roles in metabolic and immune system related to the health of host. This study applied non-invasive sampling and 16S rDNA high-throughput sequencing to study the gut microbiota structure of red pandas (*Ailurus fulgens*) for the first time under different geographical latitudes in captivity. The results showed that the two predominant phyla Firmicutes (59.30%) and Proteobacteria (38.58%) constituted 97.88% of the total microbiota in all the fecal samples from north group (red pandas from Tianjin Zoo and Jinan Zoo) and south group (red pandas from Nanjing Hongshan Forest Zoo). The relative abundance of Cyanobacteria in north group was significantly higher than that in south group. At the genus level, *Escherichia-Shigella* (24.82%) and *Clostridium_sensu_stricto_1* (23.00%) were common dominant genera. The relative abundance of *norank_f__norank_o__Chloroplast, Terrisporobacter* and *Anaeroplasma* from south group was significantly higher than that of north group. Alpha and Beta analysis consistently showed significant differences between north group and south group, however, the main functions of intestinal microbiota were basically the same, which play an important role in metabolic pathways, biosynthesis of secondary metabolites, microbial metabolism in different environments, and amino acid biosynthesis. The variations in gut microbiota between the northern and southern populations of the same species, both kept in captivity, which are primarily driven by significant differences in climate and diet. These findings provide a deeper understanding of the gut microbiota in red pandas and have important implications for their conservation, particularly in optimizing diet and environmental conditions in captivity.

## 1 Introduction

Gut microbiota is closely related to the health status of the host (Thacher et al., 2023). It can help the host obtain nutrition and energy from food (Flint et al., 2012) and participate in the regulation of host metabolism (Morris et al., 2017). Some gut microbiome are harmful to the host and may cause diseases (Toprak et al., 2006). The study on wildlife intestinal flora is helpful to both scientific monitoring of its natural population and *ex situ* conservation of its artificial populations (Li et al., 2023). The high-throughput DNA-sequencing technology has enhanced our understanding of gut microbiome and current studies showed that the structure of animals' gut microbiota could be affected by various factors, such as host phylogeny (McKenney et al., 2018; Laviad-Shitrit et al., 2019), living environments (Song et al., 2017), and gender (Yan et al., 2022). For instance, McKenney et al. (2018) found the structure of gut microbiota from black-headed gulls (*Chroicocephalus ridibundus*) fundamentally deviated from that of three other waterbird species, which means a correlation between the waterbird species' phylogeny and their intestine microbial community. Laviad-Shitrit et al. (2019) found that the gut microbiota of red panda (*Ailurus fulgens*) fundamentally deviated from that of three bamboo specialists, which means host phylogeny is found to shape the class of highly abundant taxa and phylogeny predominantly governs high-level microbiome community structure. As for the living conditions, it has been found that there were significant differences on species diversity and richness of intestinal flora from Asiatic black bears (*Ursus thibetanus*) living in different provinces, thus, it suggested that geography could affect gut microbiota of animals (Song et al., 2017). The abundance of Firmicutes from gut microbiota of wild long-tailed macaques (*Macaca fascicularis*) was higher than that from captive ones (Sawaswong et al., 2021). These findings indicated that the changes of the environmental exposure and types of diet could alter gut microbiota diversity and abundance in animals.

The red panda (*Ailurus fulgens*) belongs to Ailuridae family within the Carnivora order, and is classified as Endangered (EN) level on the IUCN Red List (Glatston et al., 2015). The natural population of *A. fulgens* is mainly distributed in China, Bhutan, India, Myanmar, and Nepal. Now they are listed as the national second-class protected animals in China. To date, the comparative study on gut microbiota of *A. fulgens* is relatively limited, and mainly focus on the impact of living conditions (Kong et al., 2014), age periods (Williams et al., 2018a,b), seasons (Kong et al., 2014; Williams et al., 2023), and interspecific comparisons (Huang et al., 2021). At present, there are few studies related to the impact of geographical latitude on intestinal flora structure of *A. fulgens*. Analysis of the research on the intestinal flora of red pandas shows that the high similarity of the intestinal microbiota between red pandas and giant pandas is driven by their similar diets (Huang et al., 2021), while the intestinal microbiota of red pandas also significantly differs from that of giant pandas (*Ailuropoda melanoleuca*) and black bears (Li et al., 2015). In addition, there are also many studies focusing on different parts of the intestinal composition, such as Li et al. (2015) using DGGE analysis of the bacterial diversity in the gastrointestinal tract of deceased giant pandas using the 16S rRNA gene V3 hypervariable region of bacteria (Li et al., 2015). Zeng et al. (2018a,b) used high-throughput sequencing of the V4 hypervariable region of the bacterial 16S rRNA gene to find that the bacterial diversity in the stomach and duodenum of giant pandas is low.

Therefore, in addition to comparisons with other species and differences between different parts of the body, in this study, the composition of gut microbiota from captive red pandas under different geographical latitudes were analyzed based on 16S rDNA high-throughput sequencing technology, in order to explore the effects of north-south differences on the gut flora of the same species.

## 2 Materials and methods

### 2.1 Ethics statement

This study used non-invasive sampling technology to collect fecal samples of red pandas from three zoos, and consequently did not involve hunting or direct contact with the animals.

### 2.2 Sample collection

Both Tianjin Zoo (latitude 39°04′N) and Jinan Zoo (latitude 36°42′N) are located in northern China, while Nanjing Hongshan Forest Zoo (latitude 32°06′N) is in the south. The climate in north and south China exhibits distinct variations. Due to the limited number of *A. fulgens*, only two fecal samples were collected from Tianjin Zoo, and five were collected from Jinan Zoo. Fifteen fecal samples were collected from Nanjing Hongshan Forest Zoo. Thus, a total of 22 samples from *A. fulgens* were studied and divided into two groups including north group (North01-North07) and south group (South01-South15).

These samples were collected once individual excretion behavior is observed to avoid sample repetition. The fresh fecal samples were packed into a sterile 5 ml EP tube and labeled. All samples were immediately placed on ice and taken to the laboratory as soon as possible. They were kept at −80°C until analyzed. To determine the food intake, the type of dietary food consumed were recorded (Supplementary Table 1).

### 2.3 DNA extraction and high-throughput sequencing

The total genomic DNA was extracted from fecal samples by TIANamp Stool DNA Kit (Tiangen Biochemical Technology, Beijing). The concentration and quality were determined by the Nano Drop 2000 spectrophotometer (Thermo Fisher Scientific, USA). Then, after the DNA was detected by 1% agarose gel electrophoresis, primers 338F (5′-ACTCCTACGGGAGGCAGCA-3′) and 806R (5′-GGACTACHVGGGTWTCTAAT-3′) were used to identify the16S rDNA gene V3-V4 region. The 20 μl PCR reaction system was used in this study, including 5 × FastPfu Buffer, FastPfu polymerase, 2.5 mmol/L dNTPs, template DNA, and primers (1 μmol/L), supplemented ddH_2_O to 20 μl. The cycling conditions of PCR consisted denaturation at 95°C for 3 min, 27 cycles of denaturation at 95°C for 30 s, 55°C for 30 s, and finally extension at 72°C for 45 s. The purified PCR products were sequenced at Illumina MiSeq platform by Majorbio Bio-Pharm Technology Co., Ltd. (Shanghai, China). The raw reads were deposited into the NCBI Sequence Read Archive (SRA) database under PRJNA1063518.

### 2.4 Data statistical analyses

The original sequencing data of all fecal samples were obtained by pretreatment with QIIME (V.1.9), and then the high-quality sequences were grouped into operational taxonomic units (OTUs) with a similarity of 97% by RDP classifier (version 2.11) Bayesian algorithm.

QIIME (version 1.9.1) was used to generate the taxonomic horizontal abundance (Hildebrand et al., 2014). Intestinal flora composition at class, phylum, order, family and genus level were demonstrated by community histogram. Based on the data of community abundance of each sample, Student's *t*-test was used to detect the differences between north group and south group at phylum and genus level. The heatmap was drawn using *R* language (version 3.3.1) vegan package.

Based on Mothur (version 1.30.2) virtual software, Welch's *t-*tests were used to compare the Alpha diversity indices including Ace, Chao, Shannon and Simpson index. Principal coordinates analysis (PCoA) based on unweighted UniFrac and weighted UniFrac analysis were used to explore differences in community structure between two groups in *R* language virtual software (version 3.3.1). Linear discriminant analysis (LDA) and linear discriminant analysis effect size (LEfSe) were used to evaluate the bacterial flora responsible for the differences.

In this study, the OTU abundance table was standardized by PICRUSt (version 1.1.0) software and COG function prediction of 16S rDNA sequence was performed. Kyoto encyclopedia of genes and genomes (KEGG) was selected to make prediction based on OTU data.

## 3 Results

### 3.1 Composition of microbial communities

The Illumina MiSeq 2500 sequencing run produced 1,476,472 raw reads. A total of 1,444,336 optimized sequences with an average sequence length of 419 bp were obtained in this study after the removal of low-quality reads. According to the species annotation results obtained by OTU clustering with 97% similarity, a total of 18 genera, 31 classes, 88 orders, 158 families, 307 genera, and 455 species were obtained. 32.45% shared OTUs were found in both groups. *A. fulgens* from north group had 25.73% unique OTUs, while *A. fulgens* from south group had 42.37% unique OTUs. A Venn analysis indicated that there was 306 shared OTUs in the 22 samples (Supplementary Figure 1). We further analyzed the shared and unique phylum/genus between two groups. At the phylum, 43.75% were found in both groups. *A. fulgens* from north group had no unique phylum, while those from south group had 77.78% unique phylum. A Venn analysis indicated that there was 14 shared phylum and 4 unique phyla in south group (Supplementary Figure 1). At the genus, 34.96% were found in both groups, 22.90 and 36.05% unique genus were found in north group and south group, separately. A Venn analysis indicated that there were 165 shared genera identified in all the fecal samples (Supplementary Figure 1).

**Figure 1:**
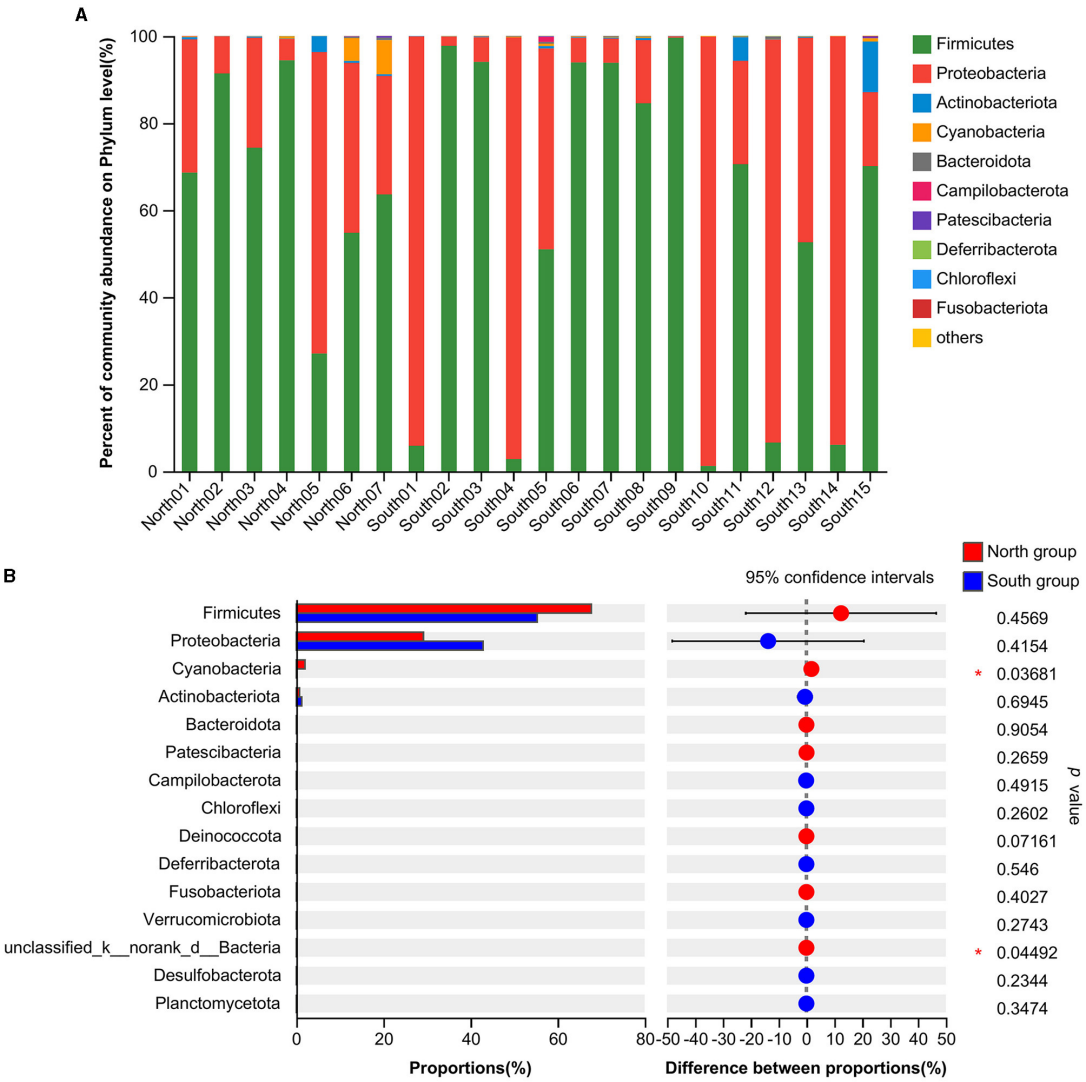
Comparison of relative abundance of microflora at phylum level **(A)** Microflora composition at phylum level; **(B)** Comparison of microflora abundance at phylum level based on Student's *t*-test, the difference between proportions represents the difference in average relative abundance between north and south groups. *p ≤ 0.05.

At the phylum level, the dominant intestinal phyla in both the north and south groups were Firmicutes (59.30%) and Proteobacteria (38.58%; Figure 1A). The common dominant bacteria of the north group included Firmicutes (67.74%), Proteobacteria (29.26%), Cyanobacteria (1.99%), Actinobacteriota (0.77%), Bacteroidota (0.13%), and Patescibacteria (0.09%). The dominant bacteria of south group mainly included Firmicutes (55.36%), Proteobacteria (42.92%), Actinobacteriota (1.27%), Cyanobacteria (0.18%), Bacteroidota (0.12%), and Campilobacterota (0.08%; Figure 1A). Firmicutes and Proteobacteria accounted for the largest proportion of dominant bacteria from both the north and south groups. The relative abundance of the Proteobacteria in south group was higher than that in north group. In contrast, the relative abundance of Firmicutes was higher in north group. According to the analysis of the top 15 phyla from two groups based on Student's *t*-tests, the relative abundance of Cyanobacteria (*p* = 0.0368) and unclassified_k__norank_d__Bacteria (*p* = 0.0449) of north group was significantly higher than that of south group. In addition, no significant differences in relative abundance of Firmicutes and Proteobacteria were found from *A. fulgens* living in the different Zoos (Figure 1B).

**Figure 2:**
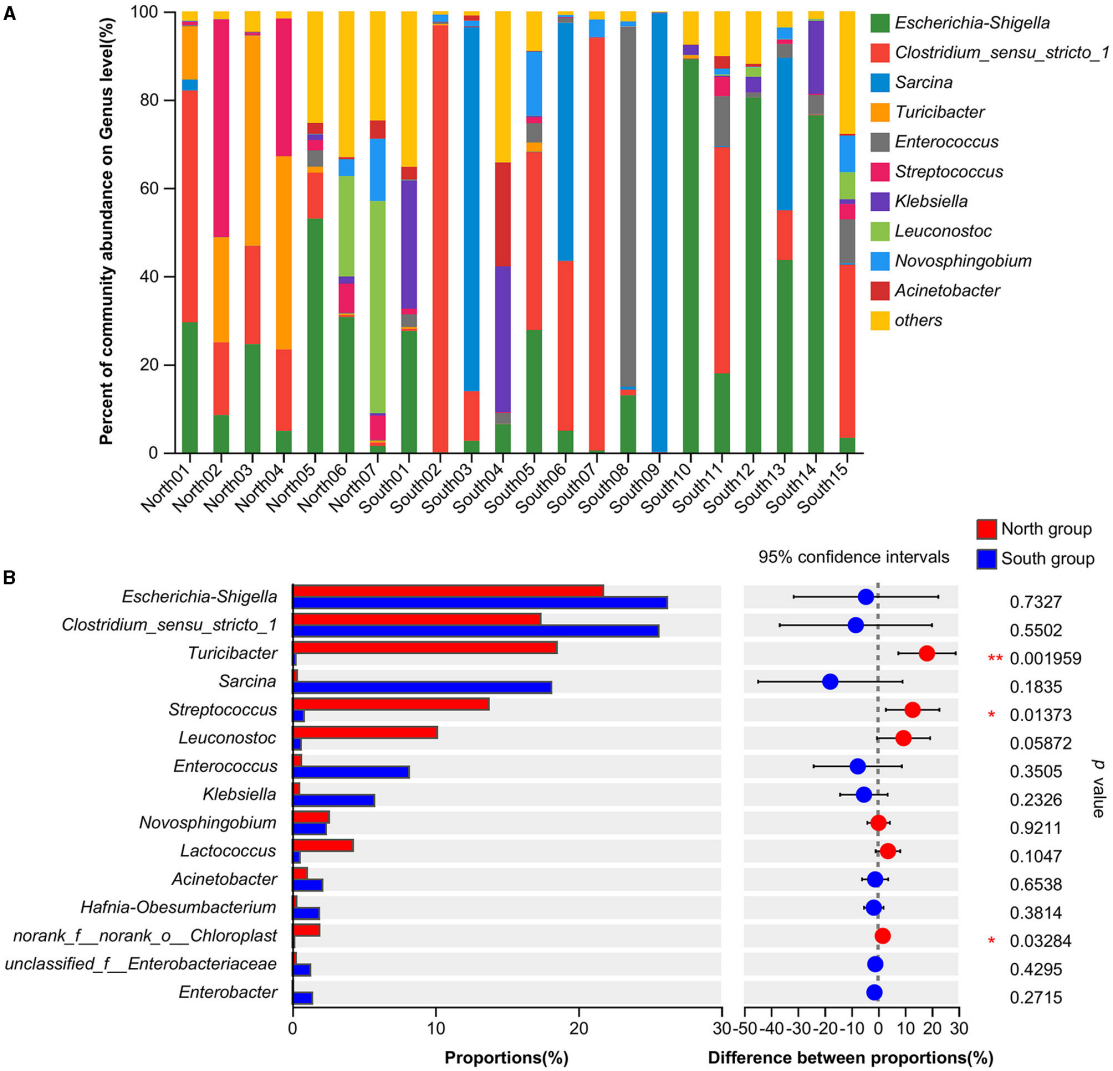
Comparison of relative abundance of microflora at genus level **(A)** Microflora composition at genus level; **(B)** Comparison of microflora abundance at genus level based on Student's *t*-test, the difference between proportions represents the difference in average relative abundance between north and south group. *p ≤ 0.05; **p ≤ 0.01.

At the genus level, the dominant bacteria genera shared by north and south groups were *Escherichia-Shigella* (24.82%), *Clostridium_sensu_stricto_1 (23.00%)*, and *Sarcina* (12.47%; Table 1). The dominant bacteria genera of north group were *Escherichia-Shigella* (21.79%), *Turicibacter* (18.50%), *Clostridium_sensu_stricto_1* (17.35%), *Streptococcus* (13.76%), *Leuconostoc* (10.13%). The dominant bacteria genera of south group were *Escherichia-Shigella* (26.24%), *Clostridium_sensu_stricto_1* (25.63%), and *Sarcina* (18.13%; Figure 2A). Based on Student's *t*-tests, the relative abundance of *norank_f__norank_o__Chloroplast, Terrisporobacter* and *Anaeroplasma* of south group was significantly higher than that of north group. However, the distribution of the dominant generas was uneven between *A. fulgens* from north group and south group (Figure 2B).

**Table 1:** Dominant phyla and genera between two groups.

**Group**	**Dominant phyla (%)**	**Dominant genera (%)**
South group	Firmicutes (55.36)	*Escherichia-Shigella* (26.24)
	Proteobacteria (42.93)	*Clostridium_sensu_stricto_1* (25.63)
		*Sarcina* (18.13)
North group	Firmicutes (67.74)	*Escherichia-Shigella* (21.79)
	Proteobacteria (29.26)	*Turicibacter* (18.50)
		*Clostridium_sensu_stricto_1* (17.35)
		*Streptococcus* (13.76)
		*Leuconostoc* (10.13)

The microbial composition from all fecal samples was also compared at the class, order, and family levels (Supplementary Figure 2).

**Figure 3:**
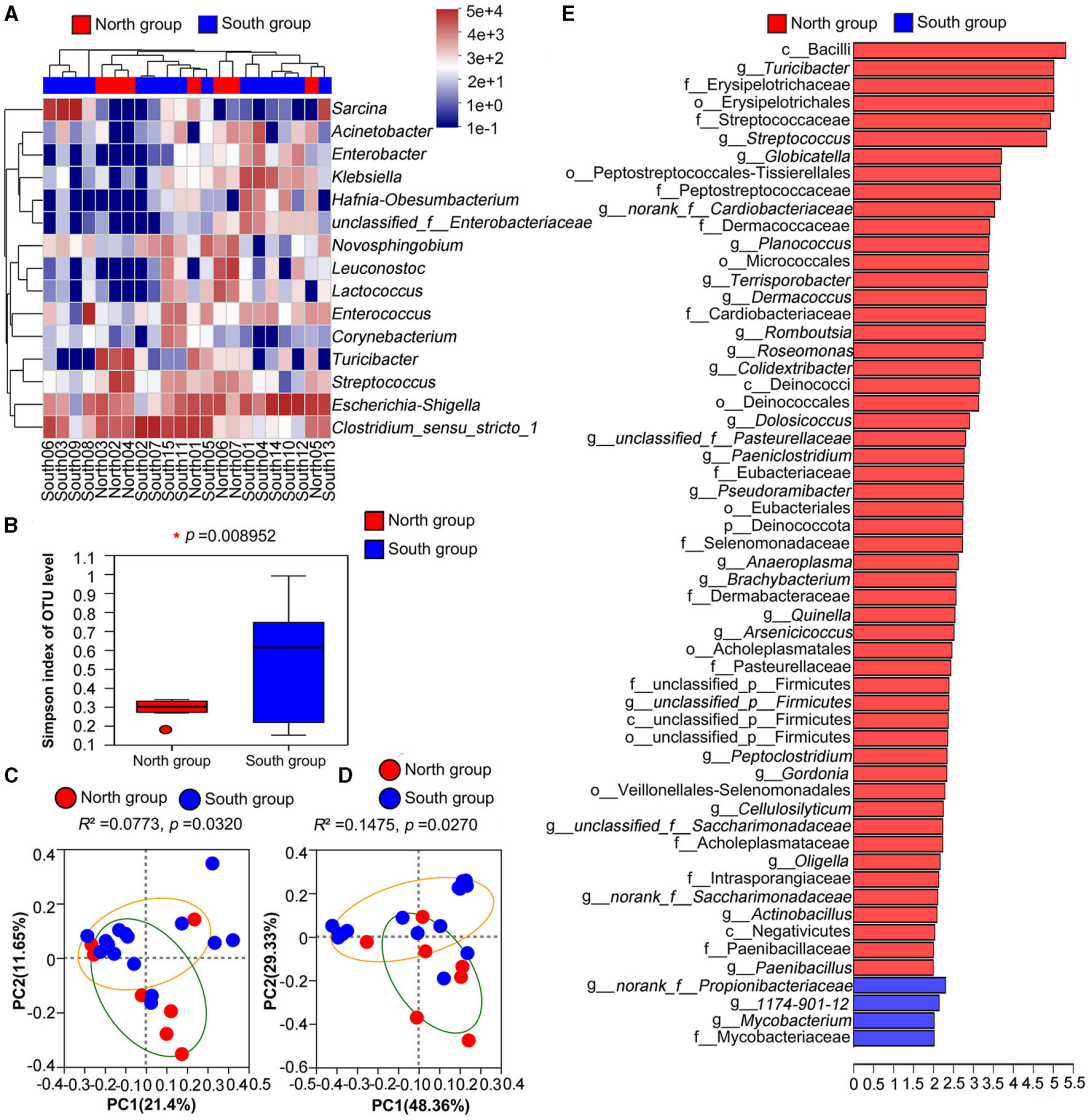
Comparative analysis between north group and south group **(A)** Heatmap of microflora in north group and south group at genus level; **(B)** Simpson index based on Welch's *t*-test; **(C)** PCoA based on Unweighted UniFrac analysis; **(D)** PCoA based on Weighted UniFrac analysis; **(E)** LEfSe analysis of microorganisms between north group and south group through linear discriminant analysis (*p* < 0.05; LDA > 2).

Clustering analysis was carried out based on the similarity of relative abundance of two groups, and presented on the community heatmap (Figure 3A). At the genus level, the north and south groups interlaced and clustered together, indicating that the two groups had a certain similarity on gut microbiota composition, but there were still large differences on the population number of related genus. LEfse analysis based on Wilcoxon rank-sum test was used, and a total of 53 and four taxa were identified in the north group and south group with the threshold of logarithmic linear discriminant analysis (LDA = 2.0), respectively. Further analysis at the genus level showed that 27 and three bacterial genera were highly enriched in the north group and south group, respectively (Figure 3).

### 3.2 Diversity comparison

From the Sobs index dilution curve and Shannon index dilution curve, it can be seen that the curve of all samples tends to be flat, indicating that the sample sequencing data in this study were large enough to reflect species information of the majority microorganisms among groups (Supplementary Figure 3). The depth of sequencing covered almost all microbial species in both sets of samples. All groups had a Coverage index of more than 99.9%, indicating the results of sequencing were reasonable.

According to Welch's *t*-tests, three indices including Ace index, Chao index and Shannon index was higher in north group than that in south group (Table 2), whereas the Simpson index of north group (0.2883) was significantly lower that of south group (0.5286). Among four Alpha indices, only Simpson index showed significant group differences, while the others showed no significant differences (Figure 3B).

**Table 2:** Mean values of Alpha diversity indices between two groups.

**Group name**	**Ace index**	**Chao index**	**Shannon index**	**Simpson index**
North group	150.03	143.92	1.68	0.28826
South group	140.28	136.92	1.23	0.52859

PCoA based on unweighted and weighted UniFrac distances were applied to determine the difference of bacterial community, and there was a significant separation between north group and south group (unweighted: *R*^2^ = 0.0773, *p* = 0.032, weighted: *R*^2^ = 0.1475, *p* = 0.0270; Figures 3C, D). Thus, PCoA analysis consistently showed significant differences between the north group and south group.

### 3.3 Functional prediction

**Figure 4:**
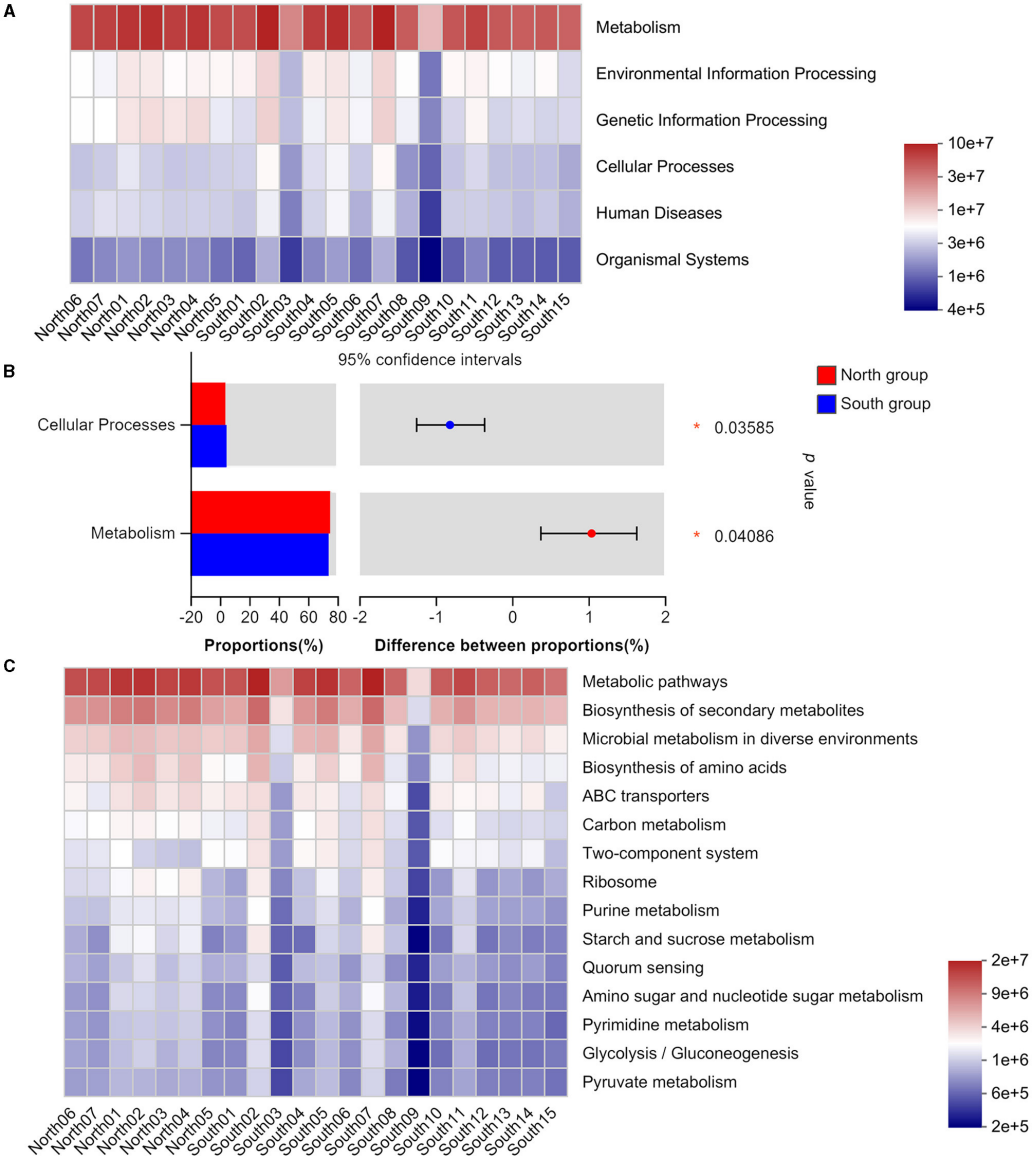
KEGG functional classification of north group and south group **(A)** Heatmap of function abundance at the KEGG Pathway Level 1. The gradient of the color block represents the change of the abundance of different functions, and the value represented by the color gradient is on the right; **(B)** Comparison of microflora functions based on Wilcoxon rank-sum test, the difference between proportions represents the difference in average relative abundance between north and south group. *p ≤ 0.05; **(C)** Heatmap of function abundance at the KEGG Pathway Level 3.

Based on Wilcoxon rank-sum test significant inter-group difference was found on the main functions of intestinal flora at the KEGG pathway level 1, including cellular processes and metabolism (Figures 4A, B). In addition, our results identified no significant differences between two groups in level 3 based on Wilcoxon rank-sum test, including those in metabolism, genetic information processing, and organismal systems. These gut microbiotas mainly play an important role in metabolic pathways, biosynthesis of secondary metabolites, microbial metabolism in different environments, and amino acid biosynthesis (Figure 4C). The functional classification analysis based on COG revealed the consistent abundance of different functions among the gut microbiota from each group (Supplementary Figure 4).

## 4 Discussion

In this study, Firmicutes and Proteobacteria were the common dominant phyla in all the fecal samples, which were basically consistent with previous studies under wild conditions (Kong et al., 2014; Li et al., 2015; Huang et al., 2021) and captive conditions (Williams et al., 2018a,b; Zeng et al., 2018a,b; Huang et al., 2021) and one closely related species, giant pandas (Wu et al., 2017). Firmicutes, which can degrade fiber and convert it into volatile fatty acids, could promote food digestion and is the main cellulose-decomposing phylum in animal intestinal tract (Jiang et al., 2021). The fermentation of dietary fiber by Firmicutes and Bacteroidetes in the intestine produces short chain fatty acids (SCFAs) such as butyric acid, propionic acid, and acetic acid, which affect host metabolism in various ways by acting on G protein coupled receptors expressed in intestinal endocrine cells (Fan and Oluf, 2020). In the guts of captive red panda individuals, Firmicutes was the most dominant phylum followed by the phyla Proteobacteria which is consistent with the previous studies (Wang, 2023). Proteobacteria, closely related to the degradation of cellulose in bamboo, can help the host utilize carbon sources and play an important role in energy accumulation (Bradley and Pollard, 2017; Yan et al., 2021). However, excessive Proteobacteria caused disease and metabolic syndrome, the level of Proteobacteria in south group was higher and the difference may related to the animal's diet.

In our study, the main food of *A. fulgens* in different zoos was bamboo. The high proportion of Firmicutes and Proteobacteria strains is conducive to the digestion of high-fiber foods, which is also common in other species with high fiber diets (Marques et al., 2017; Wu et al., 2017; McKenney et al., 2018; Senghor et al., 2018).

*A. fulgens* like to eat the leaves of bamboo, which they choose carefully. Seasonally, fruits, berries, mushrooms, bamboo shoots, and other leafy vegetation and opportunistically obtained animal protein complement the *A. fulgens*'s diet (Eriksson et al., 2010). Thus, we compared their gut composition with that of carnivorous and non-carnivorous animals. The results showed that the dominant microbiota phyla of *A. fulgens* in the present study were different from that of most carnivorous species. For example, intestinal tract of South China tiger (*Panthera tigris amoyensis*) contains a large Actinomycetes and Clostriobacteria, which could help them improve digestive problems and blood sugar control (Zhang et al., 2022). In addition, the results of *A. fulgens* were also different from those of some non-carnivorous species. For instance, the dominant phyla of chimpanzees (*Pan troglodytes*) are Firmicutes and Bacteroides (Renelies-Hamilton et al., 2019), while the dominant phyla of Indian rhinoceros (*Rhinoceros unicornis*) are Proteobacteria, Firmicutes and Bacteroidetes (Kakati et al., 2021). The potential reasons driving such differences are likely to be the difference on food composition and climate environment (Amato et al., 2013; Maurice et al., 2015; Sun et al., 2016; Trosvik et al., 2018).

*Escherichia-Shigella*, occupied 24.82% of gut bacteria, is one of the common dominant genera for both groups in this study. This taxon has also been reported as one dominant member of gut microbiota of *A. fulgens* (e.g. Williams et al., 2018a,b; Zeng et al., 2018a,b). Current evidences have found that *Escherichia-Shigella* can promote the digesting and absorbing of food by wild animals (Zeng et al., 2018a,b; Park et al., 2023), and the higher contents of fiber in animal food, the higher relative content of *Escherichia-Shigella* was found in their gut flora (Li et al., 2023). According to previous research, the change in the ratio of *Escherichia-Shigella* is related to microecological imbalance and gastrointestinal discomfort in animals, and seasonal dramatic change on its abundance was found in this species (Williams et al., 2023). However, in our study, as a harmless member of the gut microbiota of red pandas, the variation of *Escherichia-Shigella* is not significant, indicating that differences in different dimensions may have less impact on this bacterium than differences between seasons (Williams et al., 2016, 2018a,b).

The dominant microbiota genus of wild *A. fulgens* in Fengtongzhai National Nature Reserve was *Clostridiaceae unclassified*, and the relative proportion of *Escherichia-Shigella* was relatively low (Kong et al., 2014). This is probably related to the fact that the diet of wild *A. fulgens* in Fengtongzhai National Nature Reserve is mainly cellulose-rich bamboo and less artificial food. In addition, the relative content of *Bacteroides* from captive *A. fulgens* in Chengdu Zoo ranked the second dominant intestinal flora (Zeng et al., 2018a,b), while the relative content of *Bacteroides* in our study was not listed within the top 10 genus, which may be caused by the high proportion of feed and fruit in the diet in Chengdu Zoo. *Bacteroides* has the biological function of degrading proteins and carbohydrates (Bensch et al., 2023). *Clostridium_sensu_stricto_1* was another common dominant genus in this study, related with cellulose digestion (Williams et al., 2013). It could be found in fecal microorganism from not only Carnivorous orders including Amur tiger (*Panthera tigris altaica*) (Zhang et al., 2022) and Japanese badger (*Meles anakuma*) (Kaneko et al., 2023), but also non-Carnivorous orders including Sichuan snub-nosed monkey (*Rhinopithecus roxellana*) (Zeng et al., 2022). The diet of *A. fulgens* living in zoos is rich in fiber, so the high abound of *Clostridium_sensu_stricto_1* could help *A. fulgens* adapted to the diet and living conditions in captivity.

Based on the Alpha and Beta analysis, significant difference between two groups could be found on the intestinal flora diversity. Shannon index was higher in north group than that in south group. The Shannon indices of the intestinal bacteria of captive red pandas reach the highest levels during the weaning period (milk + leaf diet) (Zhao et al., 2024). A comprehensive diet (milk, seasonal fruits, carrots, biscuits, hawthorn slices) may be the reason for a higher gut microbial diversity in north group than that in south group. In this study, the relative abundance of Cyanobacteria and unclassified_k__norank_d__Bacteria in the north group was significantly higher than the south group. Some studies have found that Cyanobacteria have the ability of nitrogen fixation and carbon fixation, and participate in complex metabolic pathways with different mechanisms (Herrero et al., 2001; Hofer, 2013; Cai et al., 2020). The *A. fulgens* in the north group eat milk powder compared with the south group, and the milk powder is rich in nitrogen-containing proteins, so the content of Cyanobacteria in the north group is significantly higher than the south group.

At the genus level, the relative abundance of *Terrisporobacter* and *Anaeroplasma* in north group were both significantly higher than that in south group. Previous studies have found that *Terrisporobacter* could produce SCFAs from protein and their abundance in the host has been positively correlated with protein ingested (Lee et al., 2022). Moreover, the main food of *A. fulgens* in north groups include milk, which are rich in protein. *Anaeroplasma* related to carbohydrate metabolism, energy metabolism and nucleotide metabolism (Chen et al., 2021). Cold conditions affect the composition of the dominant intestinal flora and metabolic function, enhancing resistance to external interference and adaptability, which is beneficial to the host health (Bestion et al., 2017; Jiang et al., 2021; Al-khlifeh et al., 2023). The climate in south group is relatively warm and humid, while the climate in north group is relatively cold. Therefore, the *A. fulgens* living in north need more energy to resist the cold environment. This factor may lead to a significantly higher contents of *Terrisporobacter* and *Anaeroplasma* genus in north group.

The intestinal structure of red pandas is inconsistent with that of herbivores and carnivores (Williams et al., 2023). In addition, in order to adapt to exclusive feeding on bamboo (high-fiber, low-nutrient), the red panda has not only optimized the composition and function of its gut microflora but also formed special gut microbiota (Zhao et al., 2024). Giant pandas with similar feeding habits also have similar gut microbiota related to cellulose metabolism (Williams et al., 2013). The functional abundances of cellulases, β-glucosidase and 1,4-β-xylosidases in the red panda's gut microbiota significantly surpassed because of high cellulose and hemicellulose contents (Huang et al., 2021). β-glucosidase phylogenetic trees are very abundant in Proteobatria (Dai et al., 2021). According to the KEGG functional prediction analysis, the most important functions of our red panda intestinal flora is Metabolic pathways including starch and sucrose metabolism. This result is consistent with Huang et al. (2021), they found metagenomic analysis revealed that the symbiotic gut microbiota of the red panda possessed high levels of microbes with starch and sucrose metabolism and vitamin B_12_ biosynthesis functions.

In addition, gut microbiota structure of captive red pandas living in the north and the south China were different at both phylum level and genus level, indicating that the diversity of microbiota is not only related to diet composition, but also affected by living environment.

## 5 Conclusion

In this study, the gut microbiota structure of captive red pandas living in the north and the south China were compared at both phylum level and genus level. The results could indicate that the diversity of microbiota is not only related to diet composition, but also affected by living environment. The different phyla and genera of bacteria were analyzed, which is helpful for further investigation on structural characteristics of the intestinal flora of *A. fulgens*, but more data is needed to determine exactly how bacteria affect metabolic levels. With further advancements in metabonomic research, we can enhance our understanding of how the intestinal flora regulates nutritional metabolism and subsequently influences the health status of animals. This work will provide a scientific reference for the comprehensive protection for this endangered species.

## Data availability statement

The original contributions presented in the study are publicly available. This data can be found at: https://www.ncbi.nlm.nih.gov/sra, accession number PRJNA1063518.

## Author contributions

WC: Data curation, Writing – original draft, Investigation, Conceptualization. XC: Data curation, Writing – original draft, Investigation, Conceptualization. YZ: Data curation, Writing – original draft, Conceptualization. HW: Writing – review & editing, Supervision, Project administration, Writing – original draft, Conceptualization. DZ: Writing – original draft, Funding acquisition, Writing – review & editing, Supervision, Project administration, Conceptualization.
