# Supplementary material for: Variation on gut microbiota diversity of endangered red pandas (Ailurus fulgens) living in captivity acrosss geographical latitudes
Source: Front Microbiol. 2024 Aug 6;15:1420305. doi: 10.3389/fmicb.2024.1420305 (PMC11333448; doi:10.3389/fmicb.2024.1420305)
Supplement: Supplementary file 1 [file Data_Sheet_1.docx]

Supplementary Material

Variation on Gut Microbiota Diversity of Endangered Red Pandas (*Ailurus fulgens*) Living at Different Geographical Latitudes

**Wenqi Chen^1†^, Xiaobing Chen^1†^, Yushuo Zhang^1^, Hong Wu^1^*, Dapeng Zhao^1*^**

*** Correspondence:** Hong Wu: skywuhong@tjnu.edu.cn, Dapeng Zhao: skyzdp@tjnu.edu.cn

# Supplementary Tables

**Supplementary Table 1.** The information of the all the samples

| **Group name** | **North group** | **South group** |
| --- | --- | --- |
| **Sampling location** | Tianjin Zoo (N=2) and Jinan Zoo (N=5) | Nanjing Hongshan Forest Zoo (N=15) |
| **Sample number** | North01-North07 | South01-South15 |
| **Collection time** | May 28, 2023 and May 13, 2023 | April 21, 2023 |
| **Food types** | Cakes, cooked carrots, eggs, apples, milk powder, bamboo  seasonal fruits, steamed buns, carrots, biscuits, hawthorn slices | Bamboo, red panda cake, apples, eggs |
| **Feeding frequency** | Twice a day | Four times a day |

# Supplementary Figures

#
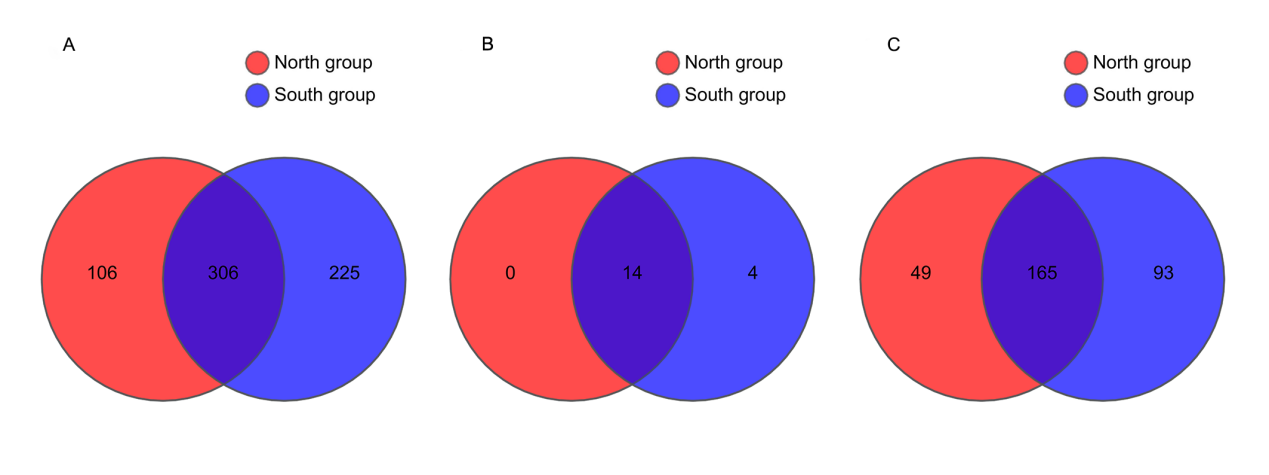


**Supplementary Figure 1.** Venn diagram of north group and south group at OTU (**A**), phylum (**B**) and genus (**C**) levels.


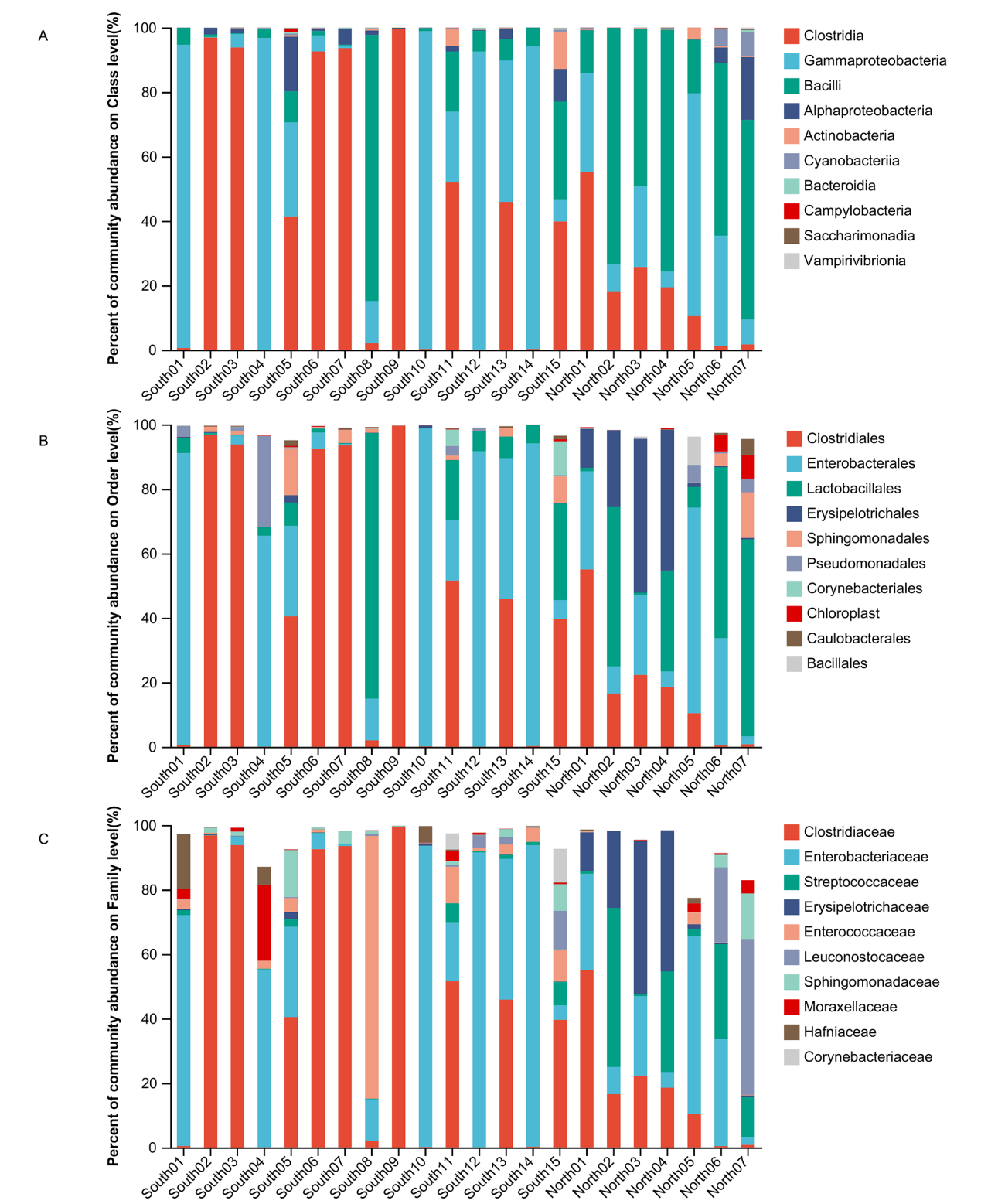


**Supplementary Figure 2.** Comparison of relative abundance of gut microflora at class (**A**), order (**B**) and family (**C**) levels.


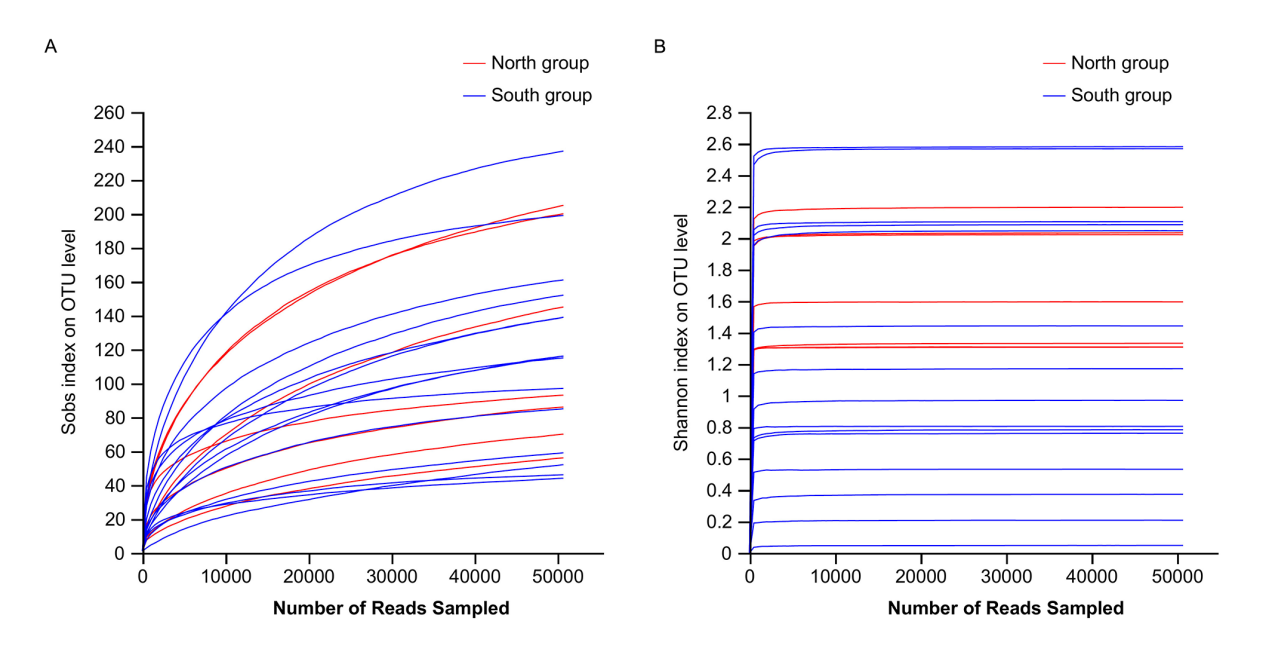


**Supplementary Figure 3.** Comparison of reads numbers based on Sobs index (**A**) and Shannon index (**B**).


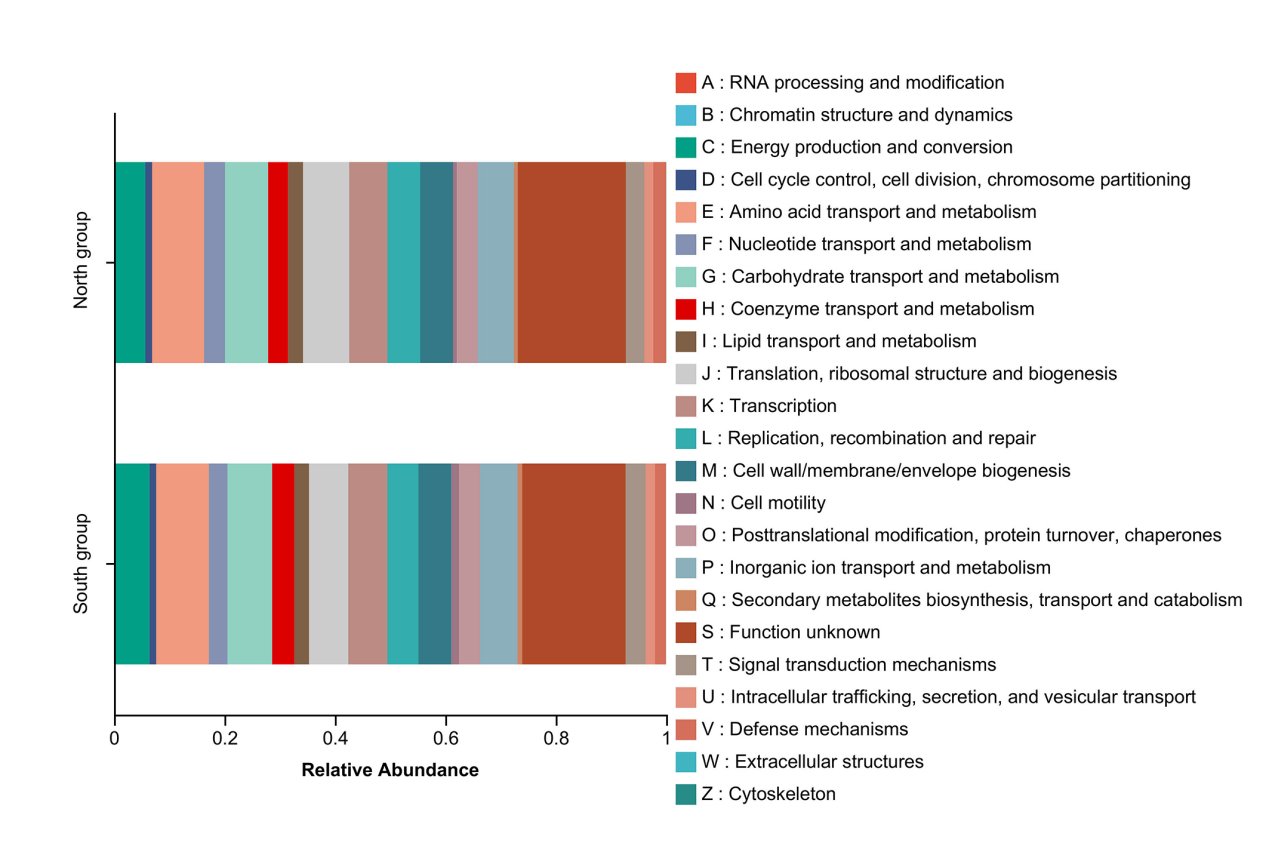


**Supplementary Figure 4.** Relative abundance of metabolic level between north group and south group.
